# Supplementary material for: Inhibition of multiple staphylococcal growth states by a small molecule that disrupts membrane fluidity and voltage
Source: mSphere. 2024 Mar 6;9(3):e00772-23. doi: 10.1128/msphere.00772-23 (PMC10964410; doi:10.1128/msphere.00772-23)
Supplement: Supplemental Figures — Supporting data for Fig. 2 and 4. [file msphere.00772-23-s0001.pdf]

Figure S1 A - E

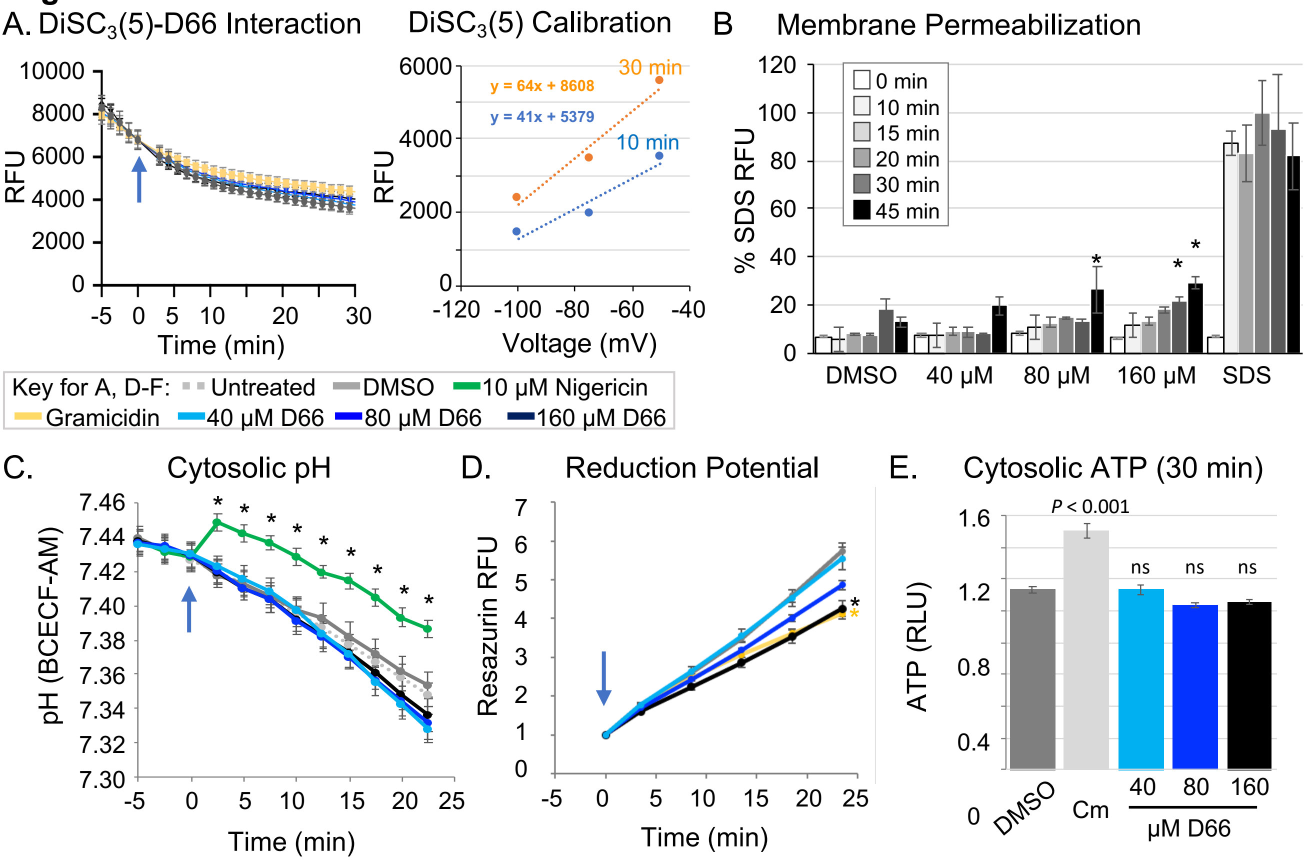

Figure S1. Supporting data for Figure 2. Mid-log phase *S. aureus* FDA209 cells were used for all experiments.

A) $DiSC_3(5)$ interaction with D66 (left) and calibration of $DiSC_3(5)$ RFU for FDA209. D66 or gramicidin (2 µg/ml; 1.1 mM) were added to $DiSC_3(5)$ just after time 0 (arrow). Data were normalized to DMSO at time 0. Relative fluorescent units (RFU). Mean and SEM of three biological replicates performed with technical triplicates. Calibration of $DiSC_3(5)$ RFU was based on potassium concentrations in the medium (see methods). The 10- and 30-minute post-treatment timepoints correspond to those in Figures S1A and 2B, C.

B) Membrane permeability was monitored by PI fluorescence. Cells were treated just after time 0 with DMSO, SDS (0.3%), or D66. Samples were processed at the timepoints shown. Mean and SEM of three biological replicates performed with technical triplicates, normalized to the highest SDS value (20-min). Asterisks indicate $P \leq 0.05$ compared to DMSO, determined by one-way ANOVA with a Tukey post-test.

C) Intracellular pH was measured with the fluorescent probe BCECF-AM. Cells were treated just after time 0 (arrow) with DMSO, the protonophore nigericin [10 µM], or D66. Mean and SEM of three biological replicates performed with technical triplicates normalized to time 0. Asterisks indicate $P < 0.0001$ compared to DMSO, determined by one-way ANOVA with a Dunnett's multiple comparison test.

D) Reduction potential (respiration) was determined with resazurin (alamar blue). Cells were treated just after time 0 (arrow) with DMSO, gramicidin (8 µg/mL), or D66. Mean and SEM of three biological replicates performed with technical triplicates normalized to time 0. Asterisks indicate $P < 0.05$ compared to DMSO, determined by one-way ANOVA with a Dunnett's multiple comparison test.

E) Membrane permeability, as monitored by ATP leakage with the Promega BacTiter-Glo kit after 30 minutes of treatment with DMSO, chloramphenicol (Cm, 32 mg/mL), or D66. Mean and SEM of relative luciferase units (RLU) for three biological replicates performed with technical triplicates and normalized to untreated cells. One-way ANOVA with a Dunnett's multiple comparison test compared to DMSO.

Figure S1 F - G

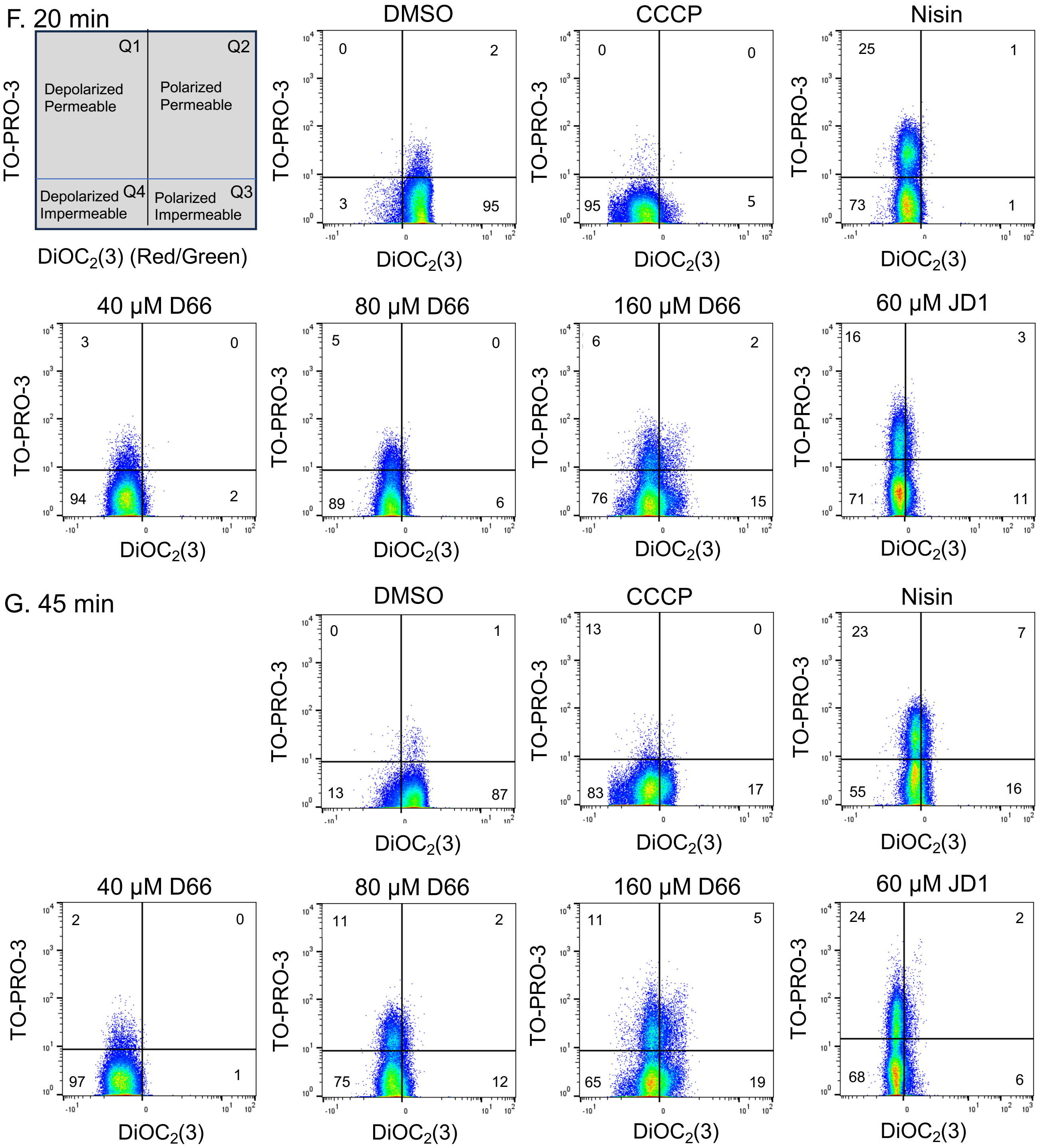

Figure S1. Supporting data for Figure 2. Mid-log phase *S. aureus* FDA209 cells were used for all experiments.

F, G) Membrane polarization and permeability were determined by flow cytometry using the fluorescent dyes TO-PRO-3 and DiOC$_2$(3). Cells were treated with DMSO, CCCP [30 µM], nisin [75 µg/mL] or D66 for 20 or 45 minutes. Data shown are representative of three biological replicates.

Figure S1 H

Percentage of cells per quadrant (Q) in Figure 2C, 5 minutes.				
Treatment	Depolarized Permeable (Q1)	Polarized Permeable (Q2)	Polarized Impermeable (Q3)	Depolarized Impermeable (Q4)
DMSO	1 +/- 1	9 +/- 9	82 +/- 9	8 +/- 3
CCCP	1 +/- 0	0 +/- 0	10 +/- 4	89 +/- 4
Nisin	28 +/- 5	3 +/- 2	6 +/ 5	63 +/- 7
40 µM D66	2 +/- 1	1 +/- 0	26 +/- 24	71 +/- 24
80 µM D66	9 +/- 1	2 +/- 0	14 +/- 1	76 +/- 1
160 µM D66	13 +/- 3	4 +/- 1	14 +/- 3	69 +/- 4
60 µM JD1	34 +/- 13	3 +/- 1	6 +/- 3	57 +/- 9

Percentage of cells per quadrant (Q) in Figure S1 F, 20 minutes.				
Treatment	Depolarized Permeable (Q1)	Polarized Permeable (Q2)	Polarized Impermeable (Q3)	Depolarized Impermeable (Q4)
DMSO	0 +/- 0	2 +/- 1	96 +/- 2	1 +/- 1
CCCP	1 +/- 1	0 +/- 0	7 +/- 2	93 +/- 3
Nisin	41 +/- 15	1 +/- 0	2 +/- 1	56 +/- 14
40 µM D66	3 +/- 1	2 +/- 1	45 +/- 18	50 +/- 20
80 µM D66	11 +/- 6	1 +/- 1	9 +/- 2	80 +/- 9
160 µM D66	10 +/- 4	2 +/- 0	12 +/- 5	77 +/- 3
60 µM JD1	27 +/- 11	9 +/- 2	27 +/- 10	37 +/- 4

Percentage of cells per quadrant (Q) in Figure S1 G, 45 minutes.				
Treatment	Depolarized Permeable (Q1)	Polarized Permeable (Q2)	Polarized Impermeable (Q3)	Depolarized Impermeable (Q4)
DMSO	0 +/- 0	3 +/- 1	92 +/- 3	4 +/- 1
CCCP	1 +/- 0	0 +/- 0	7 +/- 1	92 +/- 1
Nisin	36 +/- 8	1 +/- 0	1 +/- 0	63 +/- 8
40 µM D66	2 +/- 1	2 +/- 2	30 +/- 25	66 +/- 27
80 µM D66	9 +/- 2	1 +/- 1	10 +/- 4	79 +/- 6
160 µM D66	11 +/- 3	3 +/- 1	14 +/- 3	73 +/- 6
60 µM JD1	20 +/- 5	3 +/- 0	12 +/- 0	65 +/- 6

Figure S1. Supporting data for Figure 2. Mid-log phase *S. aureus* FDA209 cells were used for all experiments.
H) Mean and SEM of the flow cytometry data at 5, 20, and 45 minutes.

Figure S2

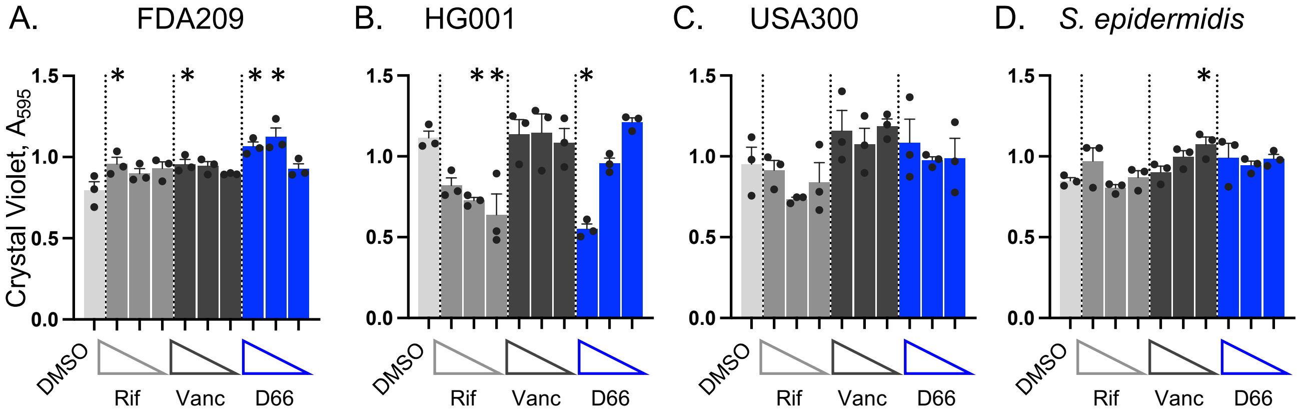

Figure S2. D66 minimally affects 5-day-old staphylococcal biofilms.

A-D). For the indicated strain, biofilms established in TSB for 5-days were treated for 18 hours with DMSO, rifampin (1x MIC_{95} = 0.05 µg/mL), vancomycin (1x MIC_{95} = 1 µg/mL), or D66 (Figure 1B). Remaining biofilm matrix was quantified with crystal violet across 3 compound concentrations (4x, 2x, and 1x MIC from left to right). Mean and SEM from three biological replicates performed in triplicate. Asterisks indicate $P \leq 0.05$ compared to DMSO, determined by one-way ANOVA with a Dunnett's post-test.
